# Comparing Biochemical and Raman Microscopy Analyses of Starch, Lipids, Polyphosphate, and Guanine Pools during the Cell Cycle of *Desmodesmus quadricauda*

**DOI:** 10.3390/cells10010062

**Published:** 2021-01-03

**Authors:** Šárka Moudříková, Ivan Nedyalkov Ivanov, Milada Vítová, Ladislav Nedbal, Vilém Zachleder, Peter Mojzeš, Kateřina Bišová

**Affiliations:** 1 Institute of Physics, Faculty of Mathematics and Physics, Charles University, Ke Karlovu 5, CZ-12116 Prague 2, Czech Republic; sarka.moudrikova@gmail.com (Š.M.); mojzes@karlov.mff.cuni.cz (P.M.); 2 Institute of Bio- and Geosciences/Plant Sciences (IBG-2), Forschungszentrum Jülich, Wilhelm-Johnen-Straße, D-52428 Jülich, Germany; l.nedbal@fz-juelich.de; 3 Laboratory of Cell Cycles of Algae, Centre Algatech, Institute of Microbiology of the Czech Academy of Sciences, Novohradská 237, CZ-37981 Třeboň, Czech Republic; ivanov@alga.cz (I.N.I.); vitova@alga.cz (M.V.); zachleder@alga.cz (V.Z.); 4 Faculty of Science, University of South Bohemia, Branišovská 1760, CZ-37005 České Budějovice, Czech Republic

**Keywords:** microalgae, *Desmodesmus quadricauda*, cell cycle, starch, lipids, polyphosphate, guanine, confocal Raman microscopy

## Abstract

Photosynthetic energy conversion and the resulting photoautotrophic growth of green algae can only occur in daylight, but DNA replication, nuclear and cellular divisions occur often during the night. With such a light/dark regime, an algal culture becomes synchronized. In this study, using synchronized cultures of the green alga *Desmodesmus quadricauda,* the dynamics of starch, lipid, polyphosphate, and guanine pools were investigated during the cell cycle by two independent methodologies; conventional biochemical analyzes of cell suspensions and confocal Raman microscopy of single algal cells. Raman microscopy reports not only on mean concentrations, but also on the distribution of pools within cells. This is more sensitive in detecting lipids than biochemical analysis, but both methods—as well as conventional fluorescence microscopy—were comparable in detecting polyphosphates. Discrepancies in the detection of starch by Raman microscopy are discussed. The power of Raman microscopy was proven to be particularly valuable in the detection of guanine, which was traceable by its unique vibrational signature. Guanine microcrystals occurred specifically at around the time of DNA replication and prior to nuclear division. Interestingly, guanine crystals co-localized with polyphosphates in the vicinity of nuclei around the time of nuclear division.

## 1. Introduction

Green algae dividing by multiple fission such as *Chlorella sp.*, *Chlamydomonas sp.* and the chlorococcal alga *Desmodesmus quadricauda*, which was chosen here as an experimental organism, grow and multiply rapidly [1], making them ideal model organisms for algal biotechnology as well as cell biology [1]. During the day, their cells grow rapidly without interruption from cell division, which occurs at night. This feature leads to natural synchronization of such algae by diurnally alternating day light. The same light/dark regime can be used in the laboratory, yielding highly synchronized cultures composed of uniformly aged and sized cells at the same phase of life and cell cycle. Thus, culture synchronization facilitates studies of suspensions of algal cells that are all in well-defined phases of their life and cell cycles.

**Figure 1:**
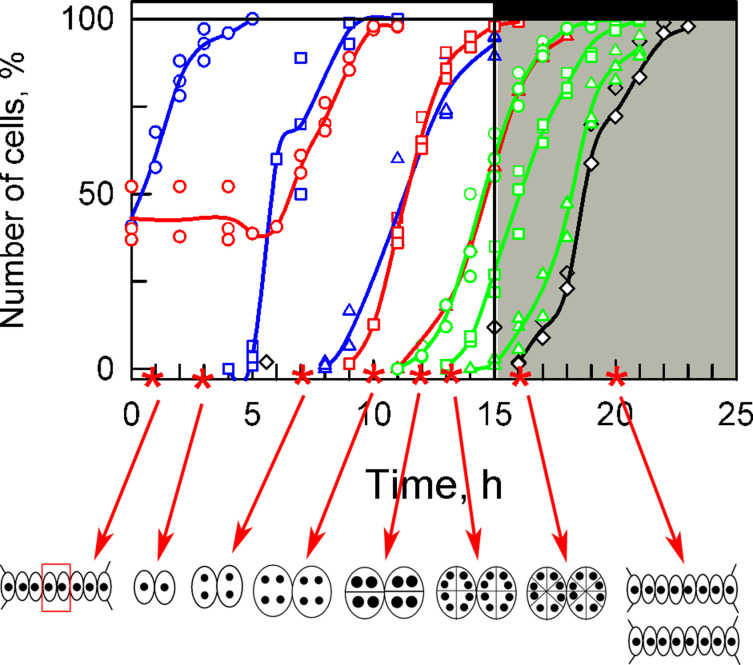
Dynamics of growth and the cell cycle in synchronized cultures of *D. quadricauda*. Upper part: Time courses of individual commitment points, nuclear division, protoplast fission and daughter cell release. Blue lines: cumulative percentage of cells that attained the commitment point for the first (circles), second (squares) and third (triangles) reproductive sequences, respectively; red lines: cumulative percentage of cells, in which the first (circles), second (squares), and third (triangles) nuclear divisions were terminated; green lines: cumulative percentage of cells, in which the first (circles), second (squares) and third (triangles) cell divisions were terminated, respectively; black line, empty diamonds: percentage of cells that released daughter coenobia. Light (15 h) and dark periods (9 h) are marked by stripes above panels and separated by vertical lines. The lines represent the means of at least three independent experiments. The raw values are plotted as dots and the line connects the mean values of the experiments. All values were calculated per parental cell, even after their division (17:00 to 22:00 h). Lower part: Schematic representation of the cells at the time-points (denoted by red asterisks) corresponding to the set of samples analyzed by Raman microscopy in Figure 4 (full set of Raman measurements is available in Figure S5). Schematic pictures of the cells indicate changes in their sizes. The full circles inside illustrate the ploidy and number of nuclei during the cell cycle. Larger circles indicate a doubling of DNA. For more details see text. Modified after Zachleder, et al. [41], Bišová and Zachleder [42], and Zachleder, Bišová, and Vítová [1].

The cell cycle, i.e., the period between two cell divisions, consists of two parts: growth and reproduction. Each reproductive sequence is composed of DNA replication, nuclear division and cell division. Cells of most organisms divide into two daughter cells and their cell cycle consists of a single growth and reproductive sequence. The cell cycle of *D. quadricauda,* and of some other chlorococcal and volvocal algae, may consist of one or several pairs of growth and reproductive sequences, thus leading to so called multiple fission. In *D. quadricauda,* DNA replication and nuclear division of the same reproductive sequence are temporally separated by several hours. In this organism, the cells are routinely multinuclear, as cell division is represented by one to several rounds of protoplast fission, closely following each other after nuclear division of the last reproductive sequence (Figure 1). Depending on the number of growth and reproductive sequences, a single mother cell of *D. quadricauda* can produce two, four, or eight daughter cells. The daughter cells originating from one mother cell remain connected by a shared cell wall in a structure called a coenobium. The obligate coenobial growth of this species provides a convenient means of monitoring growth, by simply counting cells in a particular coenobia and noting its two-fold, four-fold, or higher multiple fission pattern. Furthermore, the coenobia of *D. quadricauda* are particularly suitable for long-term microscopic studies, including confocal Raman microscopy, since they can be easily immobilized between a microscope slide and coverslip with their two largest dimensions running parallel to the slide surface, and, consequently, cellular structures of all cells of each coenobium can be found in the same focal plane.

In green algae dividing by multiple fission, the photosynthetic assimilation of carbon dioxide supports growth of most cell structures during the light phase as well as the accumulation of energy and other reserves that are required for or distributed by cell division in the dark phase. Thus, increasing cell volume, accumulation of bulk RNA and protein content or increasing energy and carbon stores can all serve to quantify growth. Furthermore, growth is accompanied by an increase in non-carbon energy rich compounds such as polyphosphates (polyP) that can serve both as components of nucleic acids (DNA, RNA) and as an energy store. Recently, guanine crystals were identified in the algal cells by Raman microscopy [2]. Although they remain rather enigmatic, they seem to be ubiquitous among microalgae [3] and might serve either as a non-specific nitrogen depot [3] or directly as a precursor for nucleic acid synthesis. In multiple fission, cell growth is composed of several growth sequences. Each of them is completed by reaching a threshold critical size, in which the cells approximately double their original mass, and double their original RNA, protein, starch, and lipid contents. The cells then enter into their reproductive sequence by attaining commitment point [4,5], thus tightly coupling the completion of the growth sequence to the reproductive events [6]. Once committed, the reproductive sequence can be completed in darkness without external supplies of energy and carbon. At the same time, depending on growth conditions, the cells can enter another growth sequence that, once completed, would allow another reproductive sequence to occur before a new coenobium is formed. In this way, individual growth sequences consecutively follow each other whilst reproductive sequences run concurrently and overlap within one cell cycle in individual cell. Thus, DNA replication of the second reproductive sequence follows the nuclear division of the first reproductive sequence and precedes the nuclear division of the same (second) sequence but also cell division of the first (preceding) reproductive sequence (Figure 1). The number of reproductive sequence/s as well as the extent of their overlap within a single cell cycle is determined by growth rate, which is governed by environmental conditions so that at higher growth rates, more reproductive sequences are started [4,5,7,8,9,10,11]. Under favorable conditions, *D. quadricauda* cells can consecutively replicate DNA in as many as three separate rounds and divide their nuclei, becoming bi-, tetra-, and octonuclear (Figure 1) [4,7,12,13,14].

Coordination between growth and reproductive sequences takes place within a short time period, commitment point, when, by reaching a critical threshold, cell size is translated into entry into the reproductive sequence. To better understand how entry of the reproductive sequence and its progression are dependent on the reserves of energy, carbon, other major biogenic elements, and essential biomolecules, it is necessary to quantify those reserves in different phases of the cell cycle, preferably their mutual relationships, spatial contexts, and temporal evolution. At the macroscopic level of the cell suspension, conventional analytical methods to study the algal biomass can be used [1,8]. To analyze spatial and temporal distribution of different macromolecules at the level of individual cells, the biomolecules can be stained or fluorescently labeled and visualized by means of optical and fluorescence microscopy [15,16,17]. However, when applying standard approaches, each biomolecular compound is usually labeled and monitored individually. Consequently, information about spatial and temporal relationships between the different substances at the level of individual cells is averaged and lost.

Raman microscopy is a label-free method that combines the advantages of molecular specificity of Raman spectroscopy with a high spatial resolution of optical confocal microscopy [18]. Raman microscopy has been widely used in biology in recent years [19], because it is relatively simple to use, with no need to introduce artificial staining or labelling to the sample, and it is able to detect several chemical components simultaneously, largely non-destructively and with a sub-cellular resolution. In microalgae, extensive use of Raman microscopy has long been hampered by strong autofluorescence of the photosynthetic apparatus, which reaches intensities several orders of magnitude higher than the desired Raman signal. This narrowed the applicability of Raman microscopy to studies of carotenoids that exhibit a resonantly enhanced Raman signal [20,21,22], or to algal cells with high levels of neutral lipids [23,24,25].

To observe a conventional Stokes Raman signal of other biomolecules such as starch, proteins, and polyP, high power in-focus photobleaching has been commonly used to remove the fluorescent background [26,27]. However, in-focus photobleaching greatly slows the acquisition of Raman maps and often results in thermal decomposition and burning of the inspected cells. We have recently demonstrated that wide-area, low-intensity photobleaching of a whole algal cell (or even multiple cells at once) by a defocused laser beam shortens the time needed for photobleaching to several seconds per cell, and enables one to reliably observe and identify starch, lipid, and polyP granules within individual microalgal cells, with sub-cellular spatial resolution [28]. The analytical strength of Raman microscopy has been further demonstrated by identifying hitherto unknown guanine microcrystals in two species of microalgae [2].

In this paper, we analyzed the dynamics of starch, lipid, polyP, and guanine pools during the cell cycle of *D. quadricauda* by simultaneously using biochemical analysis of a synchronized algal suspension, and by conventional fluorescent and Raman microscopy of individual algal cells from a synchronized algal culture. The power of Raman imaging was demonstrated in *D. quadricauda* cells by identifying guanine microcrystals occurring close to dividing nuclei in synchrony with polyP grains.

## 2. Materials and Methods

### 2.1. Experimental Organism

The chlorococcal alga *Desmodesmus quadricauda* was obtained from the Culture Collection of Autotrophic Organisms, Institute of Botany (CCALA, Czech Academy of Sciences, Třeboň, Czech Republic).

### 2.2. Algae Cultivation and Synchronization of Cultures

The algal cultures were routinely (every 3–4 weeks) sub-cultured on agar plates cultivated at 25 °C in continuous light with an incident light intensity at the surface of the Petri dishes of 150 μmol (photons)·m^−2^·s^−1^ of photosynthetically active radiation from a panel of fluorescent tubes. Such stock cultures were grown for 5–7 days and then stored at 15 °C with dim light until used or sub-cultured. To prepare synchronized cultures, the microalgae were inoculated either from an agar plate or from a liquid culture and cultivated in a glass tube (3 cm diameter) placed in a temperature-controlled water bath at 30 °C. Cultivation medium according to Zachleder and Šetlík [29] was used. The culture was aerated with air enriched to 2% CO_2_ (flow rate 350 mL·min^−1^). The glass tubes were exposed to an incident light intensity of 500 μmol (photons)·m^−2^·s^−1^ of photosynthetically active radiation from a panel of fluorescent tubes for biochemical experiments and to an incident light intensity of 150 μmol (photons)·m^−2^·s^−1^ from a panel of warm-white light-emitting diodes for Raman experiments.

For synchronization, the cultures were observed by light microscopy for two or three cycles to set the correct length of both the light and dark periods under given conditions so that the cells divided mostly into eight daughter cells (connected in eight-celled coenobia). The time for darkening the cells was when about 10% of cells started their first protoplast fission. The length of the dark period was chosen to allow all cells of the population to release their daughter cells and then the duration of the light and dark periods was kept constant. In the cultivation conditions described above, the synchronization conditions were 15/9 h light/dark cycle, which were maintained for at least two to three cycles before the experiments were initiated. The cell density at the beginning of the light period was maintained by dilution at below 1.0–1.5 × 10^6^ cells·mL^−1^ to avoid light limitation. For details on the synchronization procedure see Hlavová, et al. [30]. On the day of the experiments, the synchronized daughter cells were diluted to 1.0–1.5 × 10^6^ cells·mL^−1^ to avoid light-limitation and used as inocula for experimental cultures. For biochemical experiments, the cultures were cultivated in planar glass parallel cultivation cuvettes with a light path of 2.5 cm and inner volume 2 L, illuminated with an incident light intensity of 500 μmol (photons)·m^−2^·s^−1^ of photosynthetically active radiation provided by a panel of fluorescent tubes. For Raman experiments, the cultures were grown in 3 cm diameter glass tubes, illuminated with an incident light intensity of 150 μmol (photons)·m^−2^·s^−1^ of photosynthetically active radiation from a panel of warm-white light-emitting diodes. Due to differences in cultivations, the experimental cultures for biochemical experiments contained exclusively eight-celled coenobia while cultures for Raman experiments contained both eight- and four-celled coenobia. Only the two innermost cells of eight-celled coenobia were used for Raman mapping. The cultures were sampled hourly for biochemical experiments and as permitted for analysis in the Raman experiments (for details see below).

### 2.3. 4′,6-diamidine-2′-phenylindole Dihydrochloride (DAPI) Staining

Nuclei were stained with the fluorochrome 4′,6-diamidine-2′-phenylindole dihydrochloride (DAPI) and observed through a fluorescent microscope using the method described by Zachleder and Cepák [31] and Hlavová, Vítová, and Bišová [30]. Twenty microliters of DAPI solution (5 μg/mL in 0.25% (*w*/*v*) sucrose, 1 mM EDTA, 0.6 mM spermidine, 0.05% (*v*/*v*) mercaptoethanol, 10 mM Tris–HCl, pH 7.6) were added to the frozen cell pellet, vortexed and kept for 20–30 min in the dark at room temperature. The stained cells were observed using an Olympus microscope with 360–370 nm excitation and 420–460 emission filters.

### 2.4. Cell Size and Number

The cell suspension for Coulter counter measurements was stored frozen (−20 °C). Prior to measurement, it was allowed to thaw at room temperature and vortexed thoroughly for several minutes. According to the stage of the cell cycle, 0.4 or 0.8 mL of cell culture were diluted appropriately with ISOTON II solution to a total volume of 10 mL. Two mL of the sample (approximately 10^4^–10^5^ cells per mL in the final solution, in accordance to the Coulter counter instructions) were measured using a Multisizer 3 (Beckman Coulter, Brea, CA, USA), aperture size 100 µm. Cell number was also determined in the Bürker counting chamber (Meopta, Přerov, Czech Republic).

### 2.5. Assessment of Commitment Points and Cell Division Curves

To determine whether and how many commitment points were passed, the cells were sampled at appropriate time intervals and incubated at 30 °C in darkness. At the end of the cell cycle, the percentages of binuclear daughter cells, four- and eight-celled daughter coenobia, and undivided mother cells were estimated by light and/or fluorescence microscopy [30]. The values obtained by the assay of samples were plotted against the times of sampling. The curves are termed commitment curves. The proportion of mother cells, sporangia and daughter coenobia were determined by light microscopy in cells fixed in Lugol solution (1 g I, 5 g KI, 100 mL H_2_O) at a final concentration of 10 μL of Lugol solution per 1 mL of cell suspension. Cell division and daughter cell release curves were obtained by plotting the cumulative percentages as a function of sampling time.

### 2.6. Polyphosphate Visualization

For polyP visualization, the samples were taken at designated time points during the cell cycle. In each sample, 1 mL of cell suspension was centrifuged for 2 min at 5000× *g*, the supernatant was removed and the sample was stored at −20 °C until staining. For staining, the method of Ota, et al. [32] was used.

### 2.7. Biochemical Analyses

#### 2.7.1. Estimation of Bulk RNA, DNA, and Protein

The procedure of Wanka [33], as modified by Lukavský, et al. [34], was used for the extraction of total nucleic acids. The samples were centrifuged in 10 mL centrifuge tubes (4 min at 2800× *g*), which also served for storage of the samples. The pellet of algal cells was stored under 1 mL of ethanol at 20 °C. The algae were extracted five times with 0.2 M perchloric acid in 50% ethanol for 50 min at 20 °C and three times with an ethanol–ether mixture (3:1) at 70 °C for 10 min. Such pre-extracted samples were stored in ethanol. Total nucleic acids were extracted and hydrolyzed in 0.5 M perchloric acid at 60 °C for 5 h. After hydrolysis, concentrated perchloric acid was added to achieve a final concentration of 1 M perchloric acid in the sample. The absorbance of total nucleic acids in the supernatant was read at 260 nm (A_260_).

The light activated reaction of diphenylamine with hydrolyzed DNA, as described by Decallonne and Weyns [35] was used with the following modification [36]. The diphenylamine reagent (4% *w*/*v* diphenylamine in glacial acetic acid) was mixed with the samples of total nucleic acid extracts in a ratio of 1:1 and the mixture in the test tubes was illuminated from two sides with fluorescent lamps (Tesla Z, 40 W). The incident radiation from each side was 20 W·m^−2^. After 6 h of illumination at 40 °C, the difference between the A_600_ and A_700_ was calculated. The RNA content was calculated as the difference between total nucleic acid and DNA content.

The sediment remaining after nucleic acid extraction was used for protein determination. It was hydrolyzed in 1 N NaOH for 1 h at 70 °C. The protein concentration in the supernatant after centrifugation of the hydrolysate (15 min, 5300× *g*, room temperature) was estimated by BCA assay (Thermo Fisher) according to manufacturer’s instructions. The same procedure was carried out for the calibration curve set using different concentrations of bovine serum albumin.

#### 2.7.2. Starch Assay

Cell pellets containing approximately 2 × 10^6^ cells·mL^−1^ were harvested during the cell cycle, washed with SCE buffer (100 mM sodium citrate, 2.7 mM EDTA-Na_2_, pH 7 (citric acid)), snap frozen in liquid nitrogen and stored at −20 °C. After thawing the pellets, cells were disintegrated by adding 200 µL of distilled water and 300 µL of zirconium beads (0.7 mm in diameter) followed by vigorous vortexing for 15 min at room temperature. Depigmentation of the samples was done by adding 1 mL of 80% (*v*/*v*) ethanol to the pellet and incubating in a water bath at 68 °C for 15 min after which the samples were centrifuged for 2 min at 14,000× *g* and the supernatant was removed. The depigmentation procedure was repeated 3 or 4 times (or until the pellet was completely discolored). After that, 1 mL of porcine pancreas α-amylase (Sigma-Aldrich, Prague, Czech Republic) solution (0.5 mg·mL^−1^ *w*/*v* in 0.1 M sodium phosphate buffer (pH 6.9)) was added to each sample and they were incubated for 1 h at 37 °C. The samples were centrifuged for 2 min at 14,000× *g* and the supernatant was used for the quantification of reducing sugars through the DNSA color reaction as described by Miller [37]. In short, 500 µL of supernatant were mixed with 500 µL dinitrosalicylic acid (DNSA) solution (1% (*w*/*v*) 3,5-DNSA, 30% (*w*/*v*) potassium sodium tartrate tetrahydrate, 20% (*v*/*v*) 2 M sodium hydroxide) and incubated for 5 min at 105 °C on a heat block. Following a cooling period of 10 min at room temperature, the mixture was diluted five-fold with distilled water, after which the A_570_ values of the samples were measured. The concentration of starch was estimated through a calibration curve of potato starch (Lach-Ner, Czech Republic) digested with α-amylase.

#### 2.7.3. Total Phosphate Assay

Cell pellets containing at least 4 × 10^7^ cells, were harvested at designated time points during the cell cycle, washed with distilled water, and stored at −20 °C. After thawing, 1 mL of distilled water was added to the pellets in addition to 300 µL of zirconium beads (0.7 mm in diameter). The cells were disrupted by vigorous vortexing for 15 min at 4 °C. The samples were centrifuged for 2 min at 14,000× *g*, after which the supernatant was discarded. The pellets were washed three times with a 5% (*w*/*v*) solution of sodium hypochlorite. After the last wash, 200 µL of 0.67% (*w*/*v*) potassium persulfate were added to the pellet. The samples were finally autoclaved at 121 °C for 20 min. After cooling, 200 µL of sample were pipetted on a 96 micro well plate together with 8 µL of an ammonium molybdate tetrahydrate solution (1.2% (*w*/*v*) ammonium molybdate tetrahydrate, 4.8% (*w*/*v*) potassium antimonyl tartarate sesquihydrate, and 16% (*v*/*v*) sulfuric acid) and 2 µL of 7.2% (*w*/*v*) l-ascorbic acid. The plate was incubated for 20 min in darkness at room temperature, after which the A_880_ value for each sample was measured in a plate reader (Infinite F200, Tecan Trading AG, Männedorf, Switzerland). For quantification of total phosphate and polyP, a calibration curve was constructed using a phosphate standard for ion chromatography (Sigma-Aldrich, Prague, Czech Republic).

#### 2.7.4. Polyphosphate Assays

Samples for the estimation of polyP were collected, stored, disintegrated and depigmented similarly to the samples for total phosphates. After depigmentation with a 5% (*w/v*) solution of sodium hypochlorite, 100 µL of distilled water were added to the pellet. Following an incubation time of 5 min, the samples were centrifuged for 2 min at 14,000× *g* and the resulting supernatant was collected. The elution with distilled water was repeated twice in order to ensure the optimal extraction of polyP from the pellet. Precipitation of polyP was by addition of 1.8 mL of 100% ethanol to the collected supernatant. The samples were centrifuged for 10 min. at 14,000× *g* after which the supernatant was removed. The resulting pellet was then re-suspended in 500 µL of distilled water. The hydrolysis of polyP to orthophosphates was achieved by adding 100 µL of 4% (*w*/*v*) potassium persulfate after which the samples were autoclaved for 20 min at 121 °C. After cooling, the samples were treated and analyzed as described in the quantification of total phosphates [32].

### 2.8. Raman Analyses

#### 2.8.1. Sample Preparation

To study the content and intracellular distribution of storage biomolecules during the cell cycle using Raman microscopy, fresh living cells were needed because of problematic photobleaching of autofluorescence in chemically fixed or frozen cells. Samples from a synchronized culture of *D. quadricauda* were taken every 30–60 min, as permitted by the duration of the Raman scanning, from the start of the light phase (T = 0:00 h), considered as the beginning of the cell cycle, up to the end of the cycle (T = 24:00 h); the first sample was taken 15 min after the start of the light phase.

For Raman measurements, 0.5 mL of the cell suspension was centrifuged (2000× *g* for ca. 15 s), the supernatant was discarded and a part of the pellet was mixed with ca. 20 µL of low-gelling agarose (Sigma Aldrich, 2% solution, T = 39 °C). A few µL of the agar mixture were placed between a quartz slide and a quartz coverslip, and these were sealed with Covergrip sealant (Biotium, Hayward, CA, USA). Sample preparation took about 13 min from taking the sample to the start of the Raman measurement.

#### 2.8.2. Raman Measurement

Raman maps were acquired with a WITec alpha300 RSA confocal Raman microscope (WITec, Ulm, Germany) equipped with an oil-immersion objective UPlanFLN 100×, NA 1.30 (Olympus, Tokyo, Japan). The spectra were excited with a 532 nm laser (excitation power of ca. 20 mW at the sample). The lateral and axial resolutions of the Raman microscope (according to standard test of Raman confocality by means of silicon wafer [28,38]) were ca. 250 and 900 nm, respectively. A scanning step of 125 nm in both *x* and *y* directions (thus below the Rayleigh diffraction limit of the experimental setup) was used, with 0.1 s acquisition time per pixel. The spectra were measured immediately in the range of 220–3850 cm^−1^. This range covers characteristic (<1800 cm^−1^) as well as stretching (>2800 cm^−1^) vibrations of biomolecules and water. Prior to Raman mapping, a wide-area photobleaching of the entire algal coenobium by a defocused 532-nm laser beam was applied, as described previously, to eliminate the strong autofluorescence of chlorophyll [28]. At each sampling point, a two-dimensional Raman map of the two innermost cells of a randomly selected eight-celled coenobium was acquired.

#### 2.8.3. Data Treatment

Raman maps were treated with Project Four Plus (WITec, Germany) and our own scripts for GNU Octave [39] and MATLAB (MathWorks, Natick, MA, USA) [40]. Firstly, signals of cosmic rays were removed using automated and manual functions of Project Four Plus. Next, to compensate eventual variations in laser power and efficiency of signal collection, Raman maps of different cells were normalized to the common intensity scale as follows: the pixels of the map obviously belonging to the surrounding medium were identified automatically according to the missing band of carbon–hydrogen stretching vibrations. For each Raman map, the average spectrum of the surrounding medium was calculated and the integral intensity of the oxygen-hydrogen stretching band of water (background corrected) was used as the standard intensity for uniform normalization of Raman maps. Spectral regions with characteristic Raman bands of respective biomolecules were then selected for starch (457–507 cm^−1^, band maximum at 479 cm^−1^), lipids (2836–2869 cm^−1^, band maximum at 2854 cm^−1^), polyphosphate (1143–1190 cm^−1^, band maximum at 1159 cm^−1^), guanine (613–684 cm^−1^, band maximum at 651 cm^−1^) and the region of carbon–hydrogen stretching vibrations of all biomolecules containing C-H bonds (2795–3060 cm^−1^, band maximum at around 2935 cm^−1^). The non-Raman background was subtracted as a bisector connecting mean intensities at the last 4–10 spectral points delimiting the upper and lower ends of the regions. Spectra with high levels of the respective biomolecules and the appropriate wavenumber regions are shown in Figure S1.

For visualization of distributions of biomolecules in *D. quadricauda* cells, the intensity of each band (after background correction) was calculated by integrating the band’s area. The integral intensities in Raman maps are expressed in a color scale and, for a given biomolecule, the same scale was used in all maps. The darkest color shade corresponds to the mean intensity found in the surrounding medium and the lightest one to the maximum intensity. The only exception is the intensity of carbon–hydrogen stretching vibrations, where the darkest shade corresponds to zero.

To estimate the cellular content of respective biomolecules, pixels corresponding to both cells on each Raman map were identified using manual functions of Project Four Plus. The signals from every 2 × 2 pixels were merged to reduce the noise, and the background-corrected bands of biomolecules were integrated. For each Raman map *i*, the mean value *m_i_*, and the standard error *s_i_* of the integral intensity in the merged pixels of the surrounding medium were calculated. For each pair of cells, the integral intensities were summed for pixels where the signal was greater than *m_i_* + 7*s_i_*. Remaining pixels displaying a signal below this threshold were excluded from the summation.

## 3. Results

### 3.1. Cell Cycle Characteristics

Cultures of *D. quadricauda* were synchronized by a diurnal, alternating light/dark regime. During cultivation, cell cycle progression was analyzed hourly, as well as changes in bulk RNA, protein, DNA, starch, polyP, total phosphates, and lipid contents. These standard physiological and biochemical experiments set up a baseline, with which the results from Raman microscopy were correlated. At a given light intensity, half of the population attained the first commitment point shortly after being put into the light. By the 6th and 10th hour, more than 50% of the population attained the second and third commitment points, respectively. Attainment of each of the commitment points was followed by DNA replication, nuclear division, and cell division. Within the population, attainment of the second and third commitment points were closely followed by completing nuclear division into two and four nuclei, respectively. The third nuclear division together with the first protoplast division occurred shortly before darkening the cells at the 15th hour of the cell cycle. The cell cycle was concluded after about 22 h when all mother coenobia released daughter coenobia (Figure 1).

### 3.2. Bulk Analysis of Cell Composition

**Figure 2:**
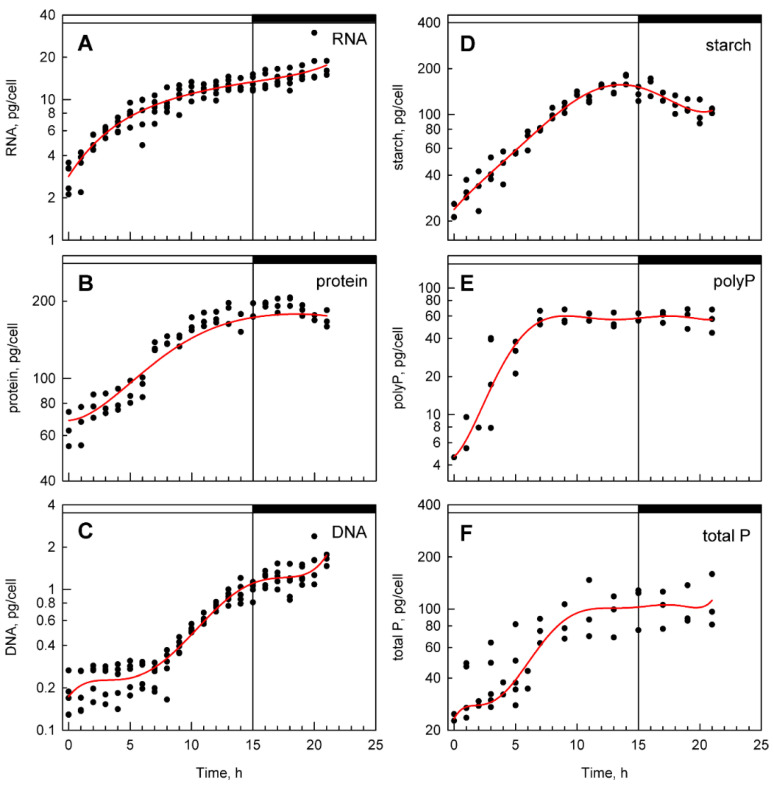
Time courses of accumulation of RNA (**A**), protein (**B**), DNA (**C**), starch (**D**), polyP (**E**), and total phosphates (**F**) in synchronized cultures of *D. quadricauda*. Light (15 h) and dark periods (7 h) are marked by stripes above panels and separated by a vertical line. Values from five different experiments are shown as dots, the smoothed red line representing the mean of five experiments in each panel. Values were calculated per parental cell even if divided (17:00 to 22:00 h).

Biochemical analysis showed that accumulation of RNA (Figure 2A) progressed rapidly in the early light period, followed by a lower rate of RNA synthesis in the late light period and in the dark. The total protein content increased with a small delay after RNA accumulation in the light and, in the dark period, protein accumulation stopped. Later in the night, the level of protein even decreased slightly (Figure 2B). A significant increase in DNA content was observed only after the 7th hour of the cell cycle, which coincides with the midpoint of the first round of nuclear division. The increase in DNA synthesis then continued until the end of the cell cycle, when the DNA content had increased about 8-fold over the initial values (Figure 2C). Starch started to be synthesized immediately after the cultures were exposed to light and continued steadily throughout the light period (Figure 2D). The rate of starch accumulation was faster until the 10th hour when the cells attained the third commitment point. It slowed from then on as the cells underwent the second and third nuclear divisions. In the dark, the starch reserves were catabolized to support nuclear and cellular divisions that were taking place in the absence of photosynthetic energy conversion.

Total phosphate and polyP content increased soon after transfer into the light (Figure 2E,F). The increase continued until about the 7th hour when DNA replication started and the first round of nuclear division was completed in half of the population. Thereafter, both total and polyP levels plateaued.

**Figure 3:**
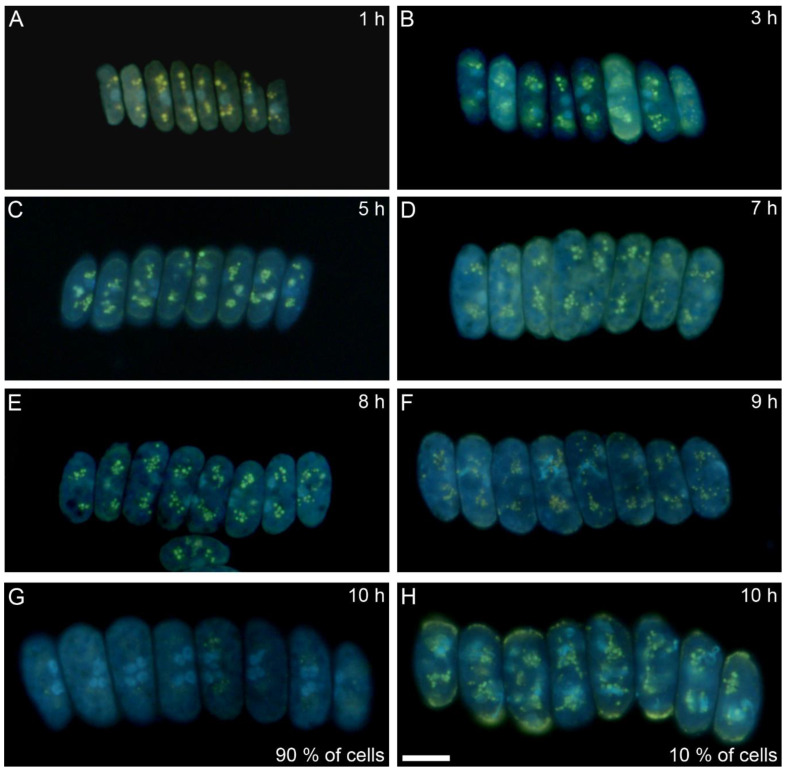
Fluorescence micrographs of coenobia stained by a high concentration of DAPI at different time points during the cell cycle. (**A**) 1st h, (**B**) 3rd h, (**C**) 5th h, (**D**) 7th h, (**E**) 8th h, (**F**) 9th h, (**G**) and (**H**) 10th h. G corresponds to situation in 90% of the cells in the synchronized population while H corresponds to situation in 10% of the cells in the synchronized population. Polyphosphates are visible as yellow spots; the bluish spots are nuclei. Bar is 10 µm.

PolyP granules can be also detected using conventional fluorescence microscopy after staining with high concentrations of DAPI [32]. PolyP granules were visible as groups of yellow spots on both sides of the bluish nuclei in the longitudinal direction (Figure 3). They were already present in the first hour of the cell cycle (Figure 3A). They seemed to multiply both in number and in extent of fluorescence in the next two hours and their localization was, conspicuously, at the outer side of two newly formed nuclei (Figure 3B). With time, both their fluorescence and number decreased (Figure 3C). By the seventh hour of the cell cycle (Figure 3D) and one hour later (Figure 3E), they were represented by groups of tiny yellow spots close to the outer cell edge in the vicinity of nuclei. From the ninth hour, the spots became even smaller (Figure 3F) and in most cells, they disappeared by the 10th hour (Figure 3G). In about 10 percent of cells, the polyP granules persisted for one more hour and were localized both to the perinuclear area and to the outer cell edge (Figure 3H). Even in these cells, the granules disappeared by the 11th hour of the cell cycle.

### 3.3. Raman Estimates of Cell Volume and Chemical Composition

Raman spectra acquired from individual pixels of the specimen consist of spectral contributions from all chemical compounds present in the respective voxels, which complicates spectral analyses. The number of contributing chemical compounds, especially in the case of dense and well-separated subcellular structures—such as lipid bodies, starch grains, polyP bodies, or crystalline inclusions—can be decreased by increasing the confocality and spatial resolution of Raman microscope. In our experiments, due to high confocality and spatial resolution reaching diffraction limit, objects separated by ca 250 nm can be resolved and Raman spectra of voxels smaller than 0.4 µm^3^ were effectively collected. This suppresses the spectral contribution of the neighboring structures. The Raman spectra within individual voxels thus contained spectral contributions of limited number of compounds and were easily interpretable by comparing them with the spectra of chemically-pure reference compounds (Figures S2 and S3). Furthermore, using advanced multivariate methods, even Raman spectra of the voxels containing a greater number of the contributing compounds can be decomposed into linearly independent spectral components corresponding to different chemically-pure species or cell structures (Figure S4).

The biochemical analyses and Raman experiments were not carried out on samples from the same cultivations because of equipment location at two remote workplaces, time and methodological constraints and different requirements on the live status of the cells. Raman microscopy was carried out immediately, using living algal cells from aliquots taken during the cell cycle, whereas biochemical analyses were carried out on samples that were accumulated and stored during the cycle.

**Figure 4:**
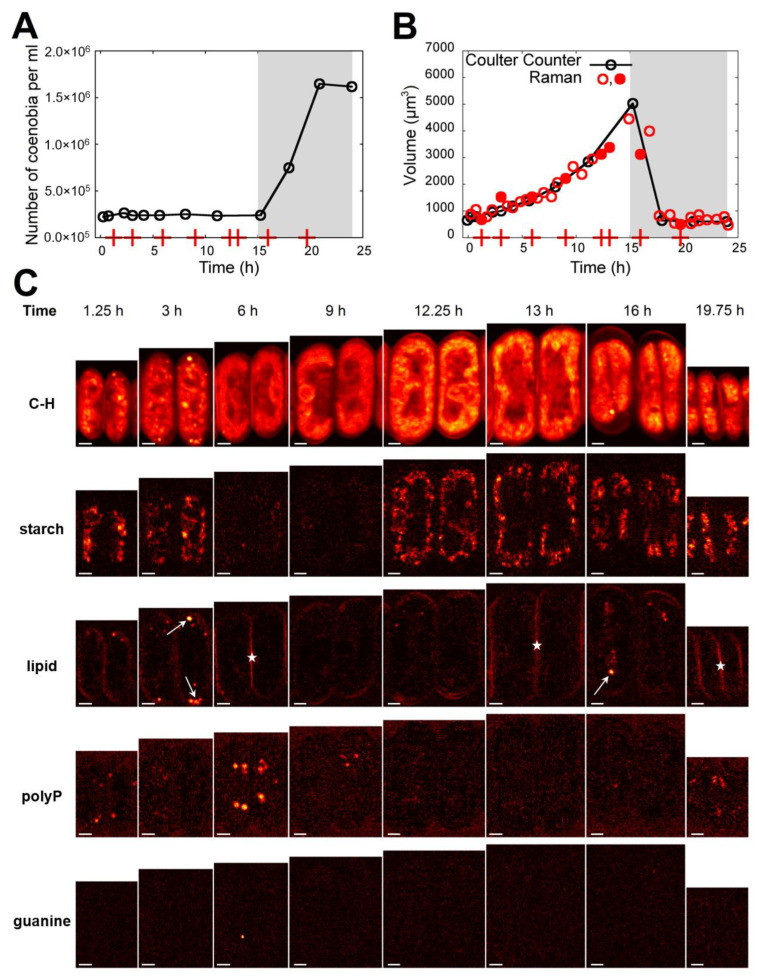
Time course of the number of coenobia in the cell culture of *D. quadricauda* as determined by Coulter counter (**A**). Time course of volumes of the coenobia determined by Coulter counter (black, median volume) and Raman microscopy (red) (**B**). For the time-point T = 18:05 h coinciding with the cell division of a majority of the cells, the Coulter counter volume of daughter cells is shown. The cells that are shown in panel (**C**) are indicated by filled circles. Raman maps showing the distribution of carbon–hydrogen (C-H) groups, starch, lipids, and polyP in the two innermost cells of eight-celled coenobia during the cell cycle (**C**). Time from start of the cycle is indicated in the top row. For a given biomolecule, the color scale is the same for all Raman maps. The white bars correspond to 2 µm. The red crosses on the time axis in panels A, B indicate times at which the cells shown in panel C were taken from the culture. The grey area in panels A, B indicates the dark period of the cycle. In panel C, lipids, the arrows depict lipid bodies, the asterisks depict membrane lipids. Spectra of both structures contain the same spectral proxy, Raman band located at ca 2854 cm^−1^.

The cultivation set-ups were the same for the two experimental methods, with the exception of light intensity, which was about three-fold lower for the Raman measurements. This led to changes in the proportion of four-celled and eight-celled coenobia within the population. The cultures used for biochemical experiments were composed exclusively of eight-celled coenobia, while the cultures for Raman measurements contained both four-celled and eight-celled coenobia. To overcome this limitation, the innermost two cells of randomly selected eight-celled coenobia were chosen for all measurements. These cells are usually the largest ones within the coenobium and the most advanced in the cell cycle. In this way, we ensured that the two analyses were carried out on as similar cells as possible. Raman microscopy mapping was performed 35-times during the 24-h cell cycle. All acquired Raman maps are presented in Figure S5. A subset of the maps is shown in Figure 4C; the age of the cells is schematically depicted in Figure 1 as well as in Figure 4A,B.

To verify the synchrony of the population used for Raman microscopy, the number of coenobia per mL of the cell suspension (Figure 4A) and median volumes of the cells (Figure 4B) were obtained by Coulter counter. To relate the bulk population analysis with the Raman microscopy results, cell volumes corresponding to the eight-celled coenobia provided by the Coulter counter measurement were compared with Raman estimates of the volume of the two innermost cells of the mapped coenobium. These estimates were calculated as follows: the number of pixels belonging to the cells, identified according to the carbon–hydrogen stretching band as described in Material and Methods, was raised to the power of 1.5 and renormalized to fit the Coulter counter values in a least squared sense. The number of pixels is directly proportional to the cross sectional area of each cell. As can be confirmed by Figure 4B, Raman estimates of the volume, although of only the two innermost cells of a randomly selected coenobium, fit well to the median volume determined for hundreds of thousands of cells by Coulter counter, indicating a good synchrony of the culture and justifying the simplifications.

For all 35 samples taken during the 24 h cycle for the Raman measurements, the distribution of starch, lipids and polyphosphate in the Raman maps were obtained by integrating the intensity of a Raman band characteristic for a given biomolecular species in each pixel spectrum, as described in Material and Methods (see also Figure S1). Additionally, the band of carbon–hydrogen (C-H) stretching vibrations was used to visualize the concentration of total organic matter (proteins, carbohydrates, lipids, RNA, DNA) in the cells.

The distributions of carbon–hydrogen stretching vibrations, starch, lipids, and polyP are shown in Figure 4C for selected time-points where the cells exhibited some characteristic features. Those particular time-points are indicated in Figure 4A,B by red crosses on the horizontal axes and their cell volumes are indicated by filled circles in Figure 4B. For all 35 samples measured here, the biomolecular distributions are shown in Figure S5.

As can be seen from Figure 4C and Figure S5, the cells already contained some starch reserves and a few lipid and polyP bodies just after the dark phase of the previous cycle. The starch content was increasing from the beginning of the light phase, reaching its first maximum at around T = 2:15 h after the light onset (Figure S5), and then declining again. The Raman band located at ca 2854 cm^−1^, which was used as spectral proxy for quantification of lipids, detects both lipid bodies and membrane lipids. The two structures are in Figure 4C, panel lipids discriminated by arrows (lipids bodies) and asterisks (membrane lipids). Lipid bodies, localized to the periphery of the cells (Figure 4C, panel lipids, arrows), increased in number and lipid content with about a 45 min delay behind the starch content. Finally, the polyP content peaked at T = 6:00 h, when the cells contained multiple polyP bodies but exhibited nearly no starch or lipid bodies identifiable by Raman microscopy.

After T = 6:00 h, the polyP bodies slowly dissolved and almost no starch or lipid bodies were visible in the cells. The carbon–hydrogen vibrations visible in interiors of the cells thus are derived predominantly from proteins (Figure 4 and Figure S5). At around T = 10:30 h, the cells started to accumulate starch again. The starch content and cell volume grew until the end of the light period at T = 15:00 h, when some of the cells were already dividing. Some lipid bodies can be seen in the cells even during cell division (Figure S5). The released daughter cells contained some starch and a few polyP bodies until the end of the experiment at T = 24:00 h. In some daughter cells, a few lipid bodies can be identified (Figure S5).

**Figure 5:**
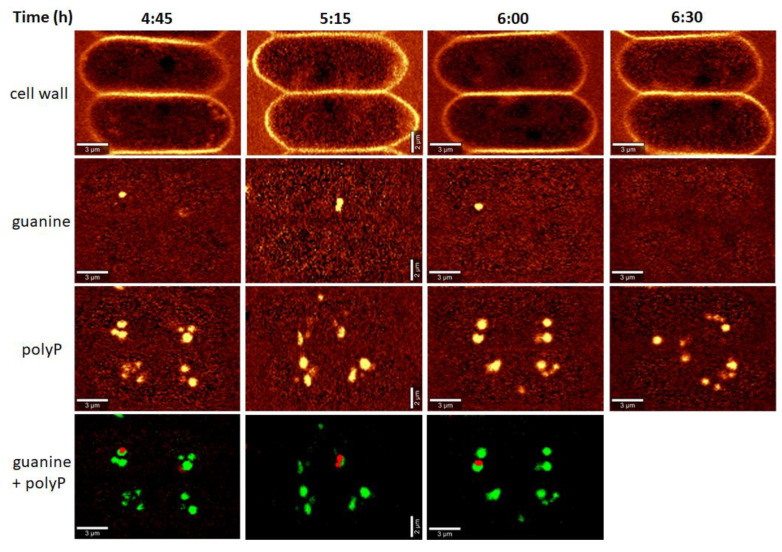
Raman maps showing the distribution of the cell walls, guanine and polyP bodies in two innermost cells of eight-celled coenobia during the early phase of the cell cycle. Time from the start of the cycle is indicated in the top row. For a given biomolecule, the color scale is the same for all Raman maps. The cell wall structure serves to visualize cell edges. It contains spectral signature of cellulose and membrane lipids, for details see Figure S4. The white bars correspond to 2 µm for Time = 5:15 h or 3 µm for other time-points. The bottom panel shows an overlay of localization of guanine (red) and polyphosphates (green).

A more detailed sampling at the early phase of the cell cycle, prior to the onset of DNA replication (between T = 4:45 h and T = 6:30 h) showed guanine bodies coinciding and possibly co-localizing with the polyP granules (Figure 5), and the cells exhibited guanine bodies between T = 4:45 h and T = 6:00 h. Small guanine bodies of a lower signal intensity were observed at T = 7:45 h, 9:45 h, and 16:00 h (Figure S5) but not in the daughter cells (Figure S5).

**Figure 6:**
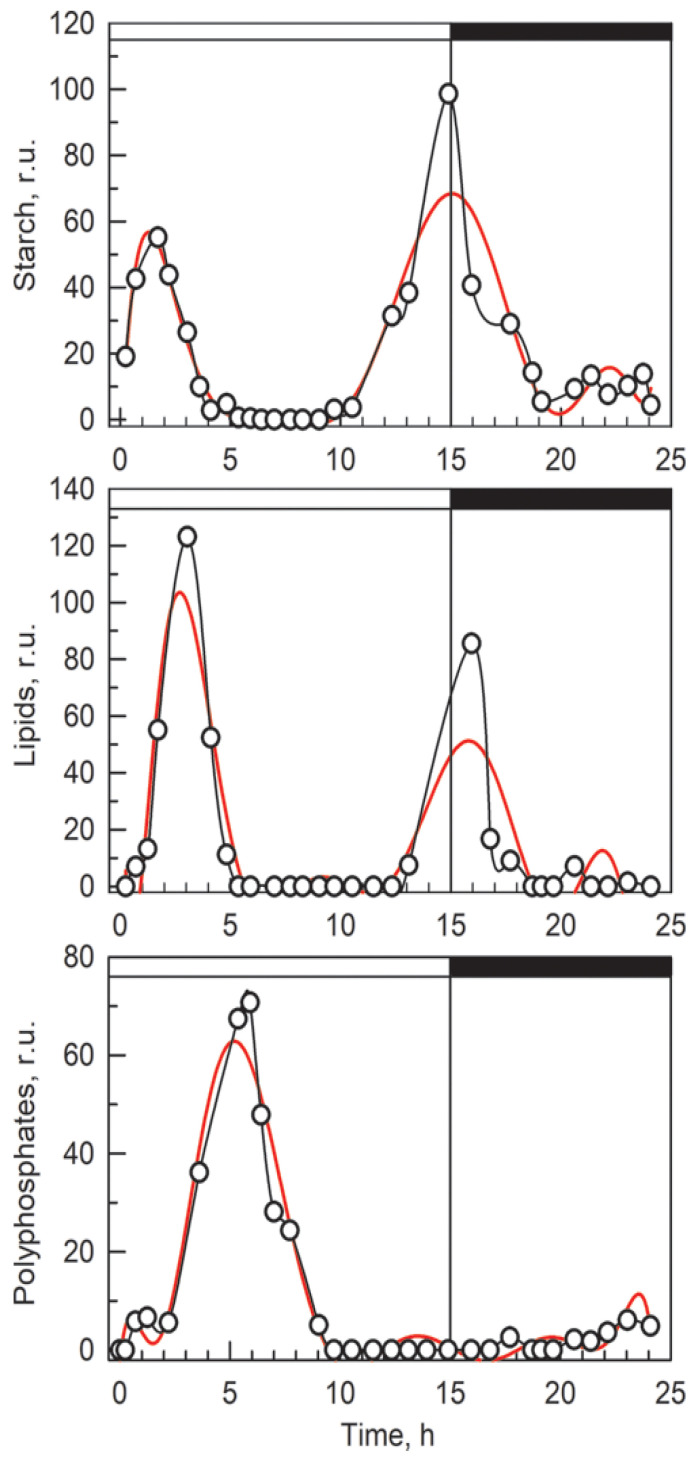
Time evolution of starch (top), lipids (middle), and polyP (bottom) levels in the cells during the cell cycle as determined from the Raman maps. Black lines connect experimental points. Red lines are splines averaging neighboring values.

To show the dynamics of the storage compounds (described qualitatively above) in a semi-quantitative way, Raman estimates of the starch, lipids and polyP content per single cell of the coenobium have been calculated by averaging contributions of both the measured cells (for details see Material and Methods) and are shown in Figure 6. The neutral lipid content was determined only from the Raman signal inside the cells. The cell’s edges (Figure 4) corresponding to membrane lipids, with the same Raman band located at ca 2854 cm^−1^ as spectral proxy, were ignored. Outlying values have been excluded for the purpose of this figure and the data points were connected with splines to guide the eye.

### 3.4. Comparison of Raman and Bulk Biochemical Analyses

**Figure 7:**
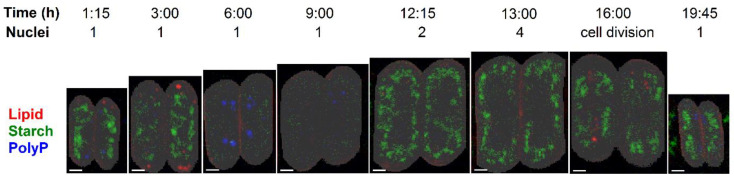
Raman maps showing the distribution of lipid droplets (red), starch bodies (green) and polyP granules (blue) in the same two innermost cells of eight-celled coenobia as presented in Figure 1C. The time counted from the beginning of the cell cycle and the number of nuclei determined by DAPI staining of the culture are indicated in the top row. For a given biomolecule, the color scale is the same for all maps. The white bars correspond to 2 µm.

We have presented the results of cell content estimates by bulk biochemical methods and by Raman mapping of individual cells in synchronous cultures. The two methods differed in the biomolecules they are were able to detect and quantify. The bulk biochemical methods could easily separate RNA, DNA, and proteins (Figure 2) due to their different chemical properties. In contrast, Raman estimates of these three macromolecules are limited by a lack of intense and specific spectral signatures as well as by a relatively low density in the mapping voxels due to a high degree of hydration. On the other hand, Raman microscopy is surprisingly effective in detecting lipid droplets (Figure 4 and Figure 7). The same size of lipid droplets is hardly detectable by conventional Nile Red staining and fluorescence microscopy. Furthermore, we have tried to measure lipid content using a fluorescent plate reader in cells stained with Nile Red. In this way, more cells are measured at the same time, thus multiplying the signal. Even then, we were unable to reliably detect any changes in lipid content. The changes detected were within a few percent of the total lipid content, and not statistically significant. Furthermore, Raman spectroscopy was able to detect microcrystalline guanine, for which there are no alternatives for reliable in situ detection.

Both bulk methods and Raman estimates were able to detect starch and polyP. The bulk biochemical analysis identified a steady increase in starch from the beginning of the cell cycle till cell darkening and the start of cell division, when starch started to be consumed. In contrast, Raman analyses detected an increase in the starch level in the first three hours of the cell cycle followed by a complete disappearance. A new increase in starch synthesis was observed by about the 12th hour of the cell cycle and reached a peak at about the 15th hour. The starch was subsequently spent for the ongoing processes of protoplast fission and cell division. Both Raman estimates and conventional fluorescence microscopy revealed similar patterns of polyP localization in both space and time. Both of the methods detected two major groups of polyP granules on each side of a single nucleus, or later, on the outer side of the two newly formed nuclei. PolyP granules gradually disappeared by the time the cells completed division of their nuclei into four. The bulk biochemical analyses detected a rapid increase in both total and polyP content from the beginning of the light period to about the fifth hour of the cell cycle. Thereafter both total and polyP levels remained unchanged.

## 4. Discussion

Synchronized cultures are a traditional tool for studying the progression of the cell cycle in different organisms. Cell cycle progression, encompassing growth, cell duplication and division events, has been routinely described by biochemical analyzes of macromolecules (RNA, protein, DNA, starch). Such analyzes are rather time consuming, complicated and mostly require a large quantity of initial sample. Here, we have compared the bulk biochemical analyses of biomolecules with the results of Raman microscopy. Raman microscopy visualizes distributions of the prevalent groups of biomolecules simultaneously, without the need for artificial pre-processing of the sample and with a spatial resolution of a confocal microscope. As Raman microscopy shows the intrinsic chemical content of the sample, even biochemical species not expected in the sample can be seen, provided they are present at sufficient concentrations and that their Raman spectra are correctly interpreted. This can be demonstrated with the example of guanine microcrystals that have been recently observed in two microalgal species [2] without prior knowledge of their existence. In the present study, the guanine microcrystals occurred regularly between approximately the fifth and seventh hours of the cell cycle, i.e., at the time of attainment of the second commitment point and completion of the first nuclear division, and then sporadically during other phases of the cell cycle. The appearance of guanine microcrystals during a natural cell cycle of unstressed cells supports the tentative biological relevance of guanine microcrystals as effective depots of purines [2]. Such biological functions may be related to their localization in the cells. Guanine crystals were localized together with polyP granules, either in the vicinity of the newly formed nuclei, or in the place where nuclei are formed. The two possibilities are impossible to separate based on Raman microscopy, as nuclear localization could not be detected in the algal cells. Both localizations would suggest guanine crystals may serve as purine deposits consumed in the formation of nuclei.

Apart from identifying novel and/or otherwise undetectable biochemical species, Raman microscopy might also be more sensitive to low levels of biochemical species that can be detected and analyzed by fluorescence microscopy and/or bulk analytical techniques. This is demonstrated by the observed presence of lipid bodies at the periphery of some cells during the cell cycle (Figure 4 and Figure 7, and Figure S5). These lipid bodies could not be observed by fluorescence microscopy or bulk fluorescent measurements, as they did not increase the cellular lipid content enough to be detected separately. Raman microscopy has been established method for detection of lipids, particularly in stressed cells with increased lipid amount [10,11,12,13]. Here, this has been extended to detection of very low lipid levels present in non-stressed cycling dividing cells. Raman microscopy possibly takes advantage of its ability to detect different compounds in dense and solidified, although small, structures.

Raman microscopy and fluorescent microscopy of polyP bodies were in agreement (compare Figure 3 and Figure 5). They visualized polyP granules both in sites where new nuclei will be formed and near them, once they divided. This was particularly true for the first nuclear division. The localization of polyP granules (together with guanine crystals) close to nuclei could suggest they function as energy and phosphate stores for nucleic acid synthesis. This localization is thus in agreement with the classical observation in green alga *Chlorella*, where polyP are preferentially used for the synthesis of DNA (and phosphoproteins), but not RNA [43,44,45]. Both microscopy methods showed a decrease in polyP granules after the eighth hour of the cell cycle, i.e., at the time when the exponential increase in DNA synthesis starts, further supporting the notion that polyP granules serve as a P source for DNA synthesis. Interestingly, at the same time, the total phosphorus and polyP levels, as determined by bulk biochemical analyses, plateaued. The biochemical method that we used for total phosphorus and polyP was non-specific, and precipitated polyP granules together with DNA and RNA. This could explain the differences between microscopy and biochemical methods. Thus, from the eighth hour, DNA is possibly synthesized at the expense of polyP granules, and it gradually forms a larger proportion of the biochemically detected polyP fraction.

The most striking difference was observed between the bulk biochemical and Raman analyses of starch. The bulk biochemical analysis showed a steady increase in starch, as has been established in the field [1,42,46], whilst Raman microscopy showed a bimodal pattern with an initial increase in starch prior to attainment of the first CP followed by starch disappearance and another increase in starch levels at the time the cells reached the third CP and completed the second nuclear division (Figure 1 and Figure 6). This was really an intriguing observation, probably caused by the difference in light intensities used for cultivation in the biochemical versus Raman experiments. Light intensity is known to affect the accumulation of starch, so that it increases with increasing light intensity [47]. At lower light intensity, less starch would be synthesized; it would be continuously spent for general metabolism and no net starch granules would be visible by Raman microscopy. Although this is a somewhat problematic discrepancy, probably caused by different cultivation conditions, we leave it here because it reveals an interesting phenomenon concerning the complete consumption of accumulated starch during the cell cycle under limited illumination, which would deserve a more detailed study. Due to its high sensitivity, Raman microscopy would be well suited for such studies.

The graphical description of the time evolution of starch, lipid and polyP content during the cell cycle in Figure 4 should be treated as a semi-quantitative proxy. Although recent studies have shown that two-dimensional mapping can be used to quantify the levels of starch [26,48], lipids [26,49], and polyP [50] in microalgal cultures, these studies have averaged the results from Raman maps of several to dozens of cells in order to obtain an accurate quantification when compared to bulk methods. On the other hand, as the cells in the present study were synchronous, variability amongst the cell cultures should be much narrower than in a common non-synchronous culture. Furthermore, the Raman signal is, by the nature of conventional Raman scattering, linearly proportional to the concentration of a compound in a given voxel [18].

The data presented here have proven that Raman microscopy is capable of detecting relatively low concentrations of storage biomolecules present in non-stressed *D*. *quadricauda* cells during a normal cell cycle. Decreasing the limit of detection to levels present in standard dividing cells represents an improvement compared to previous quantification studies [26,48,49], which mostly focused on stressed cells over-accumulating lipids or starch. Although the merits of Raman microscopy have been shown here, it still possesses limitations similar to conventional fluorescence microscopy and bulk biochemical analyses. As concerns the chemical analysis and imaging, the method is well suitable for dense and solidified, although small structures, but rather insensitive for dissolved chemical species, as it requires local concentrations of at least dozens of mM. This precludes the detection of low-concentration metabolites in solution. Furthermore, Raman mapping is of relatively low throughput, and parallelization is problematic, unless one possesses several Raman microscopes. To keep a sampling time of 30–45 min and a high spatial resolution of Raman mapping, in the present study, it was not possible to measure more than two cells of *D. quadricauda* coenobium at a given time-point. Statistically, a more sound set of cells could be acquired by compromising one or both of these requirements. However, lower spatial resolution would impair imaging and quantification of small biomolecular inclusions in Raman maps. On the other hand, increasing intervals between samplings might lead to missing important phases in the cell cycle. Otherwise, there exists the possibility of repeating the same experiment in multiplicates. However, each cell culture, although synchronous, is never exactly the same. Although the specificity of Raman spectrum for a given class of chemical compounds (e.g., lipids, carbohydrates, nucleic acids, proteins) is relatively high (Figures S2–S4), the specificity within particular classes is often much smaller. For example, structurally different proteins of similar mean amino acid composition cannot be discerned. It cannot be used for unknown molecules as their Raman signature is unknown. Furthermore, the excitation energies used for conventional, non-resonant Raman spectroscopy is several orders of magnitude higher than powers required for confocal fluorescence microscopy, which raises concerns about the local heating and photodegradation of some less stable compounds. In photosynthesizing organisms such as microalgae, high fluorescence background, in particular that of chlorophyll, represents the main obstacle for Raman microscopy. Although this issue can be solved by photobleaching prior to the Raman measurement [2,3,28,50,51,52,53] this prevents the more desirable time-lapse Raman microscopy of exactly the same cell or coenobium. On the other hand, photobleaching itself does not disqualify the results, as the consequent decay processes take longer than needed for Raman mapping [28]. Despite all the aforementioned limitations and shortcomings, a major advantage of Raman microscopy is the ability to acquire chemical maps of multiple components from a single measurement that provides a unique opportunity to visualize these structures in a mutual context (Figure 7).

## 5. Conclusions

We have compared the detection of energy-rich compounds (starch, lipid, and polyP) during the cell cycle of *Desmodesmus quadricauda* using two independent methodologies, conventional bulk biochemical analyzes and confocal Raman microscopy. The two methods were comparable in detection of polyP. Fluorescence and Raman microscopies detected the specific localization of polyP granules near the nuclei until the bi-nuclear stage. The two methods differed in detection of starch, possibly suggesting differences in starch localization and/or its solubility during the cell cycle. Raman microscopy was more sensitive in detecting lipids. It also detected guanine crystals; these were localized near polyP granules and nuclei, suggesting their possible role in processes related to nuclear division. Confocal Raman microscopy is capable of detecting even low levels of macromolecules naturally present in the cells during their vegetative development. It is especially suited for the detection of polyP, lipids, and guanine crystals within the cells. The differences in starch detection will require further experiments.

## Data Availability

All data presented in this study are available within this article or Supplementary Materials. There are no special databases associated with this manuscript.

## Supplementary Materials

The following are available online at https://www.mdpi.com/2073-4409/10/1/62/s1, Figure S1: Typical Raman spectra of the respective biomolecules detected and quantified in *D. quadricauda* cells. Figure S2: Raman spectra of starch granules and crystalline guanine obtained in situ from *D. quadricauda* compared with the respective chemical references. Figure S3: Raman spectra of small lipid droplets and polyP granules obtained in situ from *D. quadricauda* compared with the respective chemical references. Figure S4: Raman chemical maps of eight-cell coenobium of *D. quadricauda* and the spectral components obtained by a multivariate deconvolution. Figure S5: Raman chemical maps of the cells illustrating in detail the time course of changes in their molecular composition during the cell cycle.
