# Supplementary material for: Comparing Biochemical and Raman Microscopy Analyses of Starch, Lipids, Polyphosphate, and Guanine Pools during the Cell Cycle of Desmodesmus quadricauda
Source: Cells. 2021 Jan 3;10(1):62. doi: 10.3390/cells10010062 (PMC7824393; doi:10.3390/cells10010062)
Supplement: Supplementary file 1 [file cells-10-00062-s001.pdf]

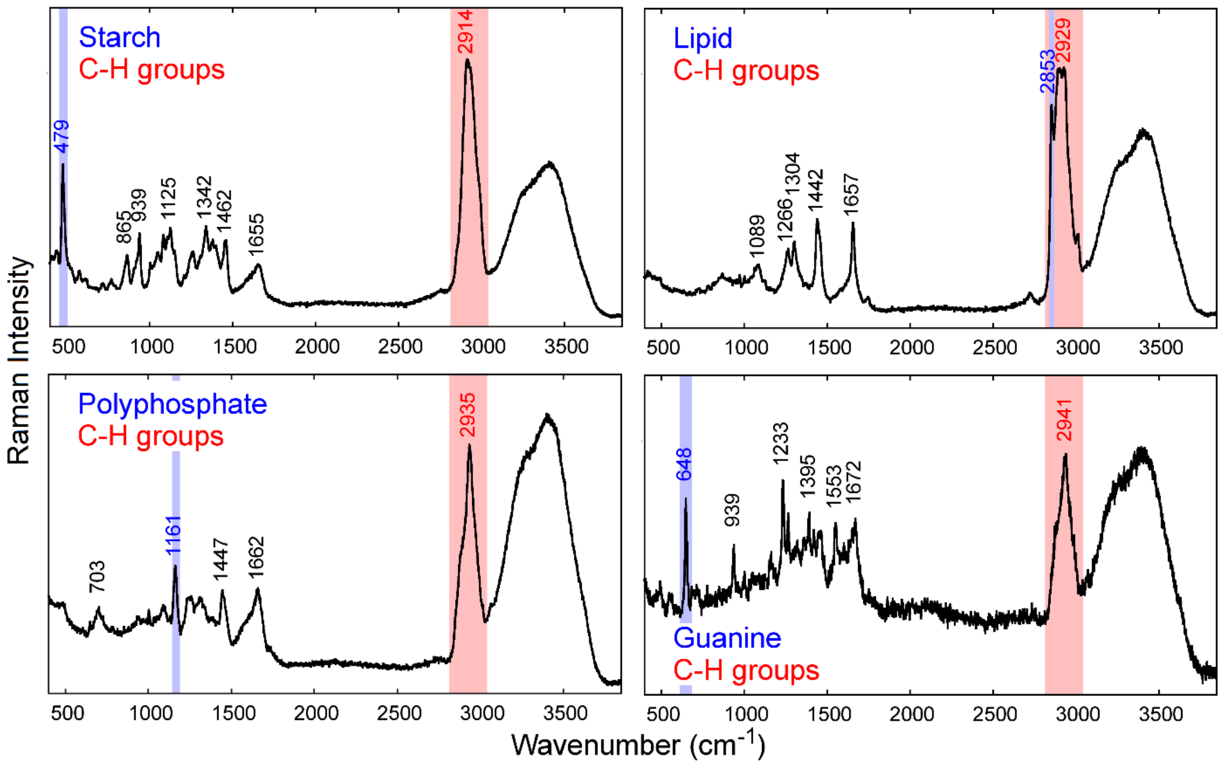

Figure S1: Raman spectra of selected pixels with prevalent contribution of starch (upper left), lipids (upper right), polyphosphate (lower left) and crystalline guanine (lower right). Spectral regions with specific bands (Raman markers) that were used for visualization and quantification of the given biomolecules are highlighted in blue. The region of stretching vibrations of carbon-hydrogen (C-H) groups was the same for all the biomolecules and it is highlighted in red. In the case of polyphosphate and microcrystalline guanine, the C-H band at ca. 2940 cm^{-1} as well as the bands at 1447 and 1662 cm^{-1}, belongs to proteins and carbohydrates co-localized with polyphosphate and guanine, since pure chemical species do not exhibit such spectral features.

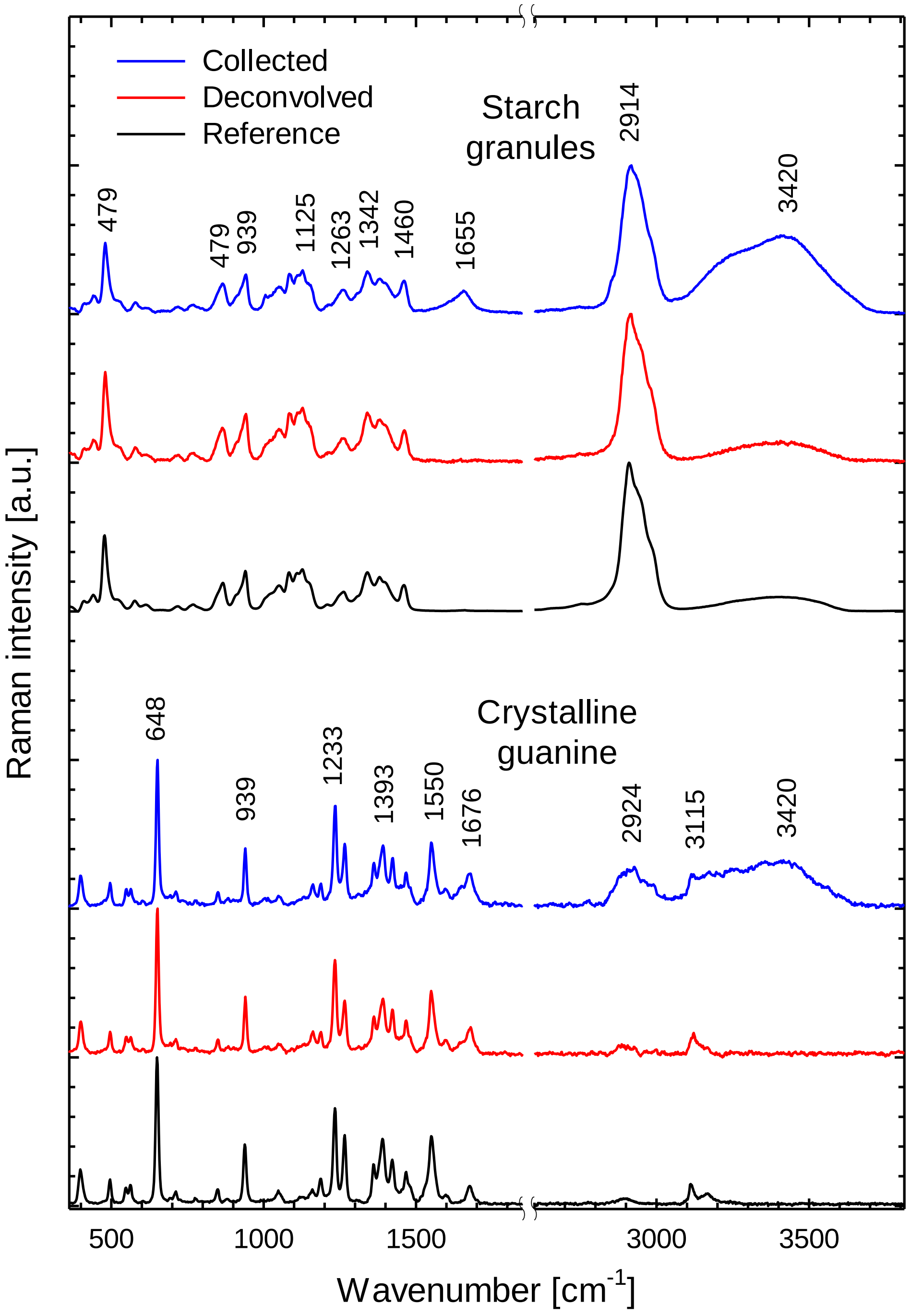

Figure S2: Typical Raman spectra of starch and crystalline guanine acquired directly from the cellular structures consisting mainly of the respective chemical compound (Collected, blue lines) compared with the Raman spectra after multivariate deconvolution (Deconvolved, red lines) and the Raman spectra of the corresponding chemically-pure references (Reference, black lines). The collected spectra exhibit also Raman features of other compounds present in the measured voxels. The broad band with a maximum at 3420 cm^{-1} belongs to the signal of omnipresent water. In the case of starch, the band at 1655 cm^{-1} can be assigned to the proteins tightly collocated with starch. In the case of crystalline guanine, the band at 2924 cm^{-1} comes probably from the lipid membrane surrounding those inclusions. Weaker Raman bands of the contributing compounds are not so clearly visible in the complex spectra, but they are present.

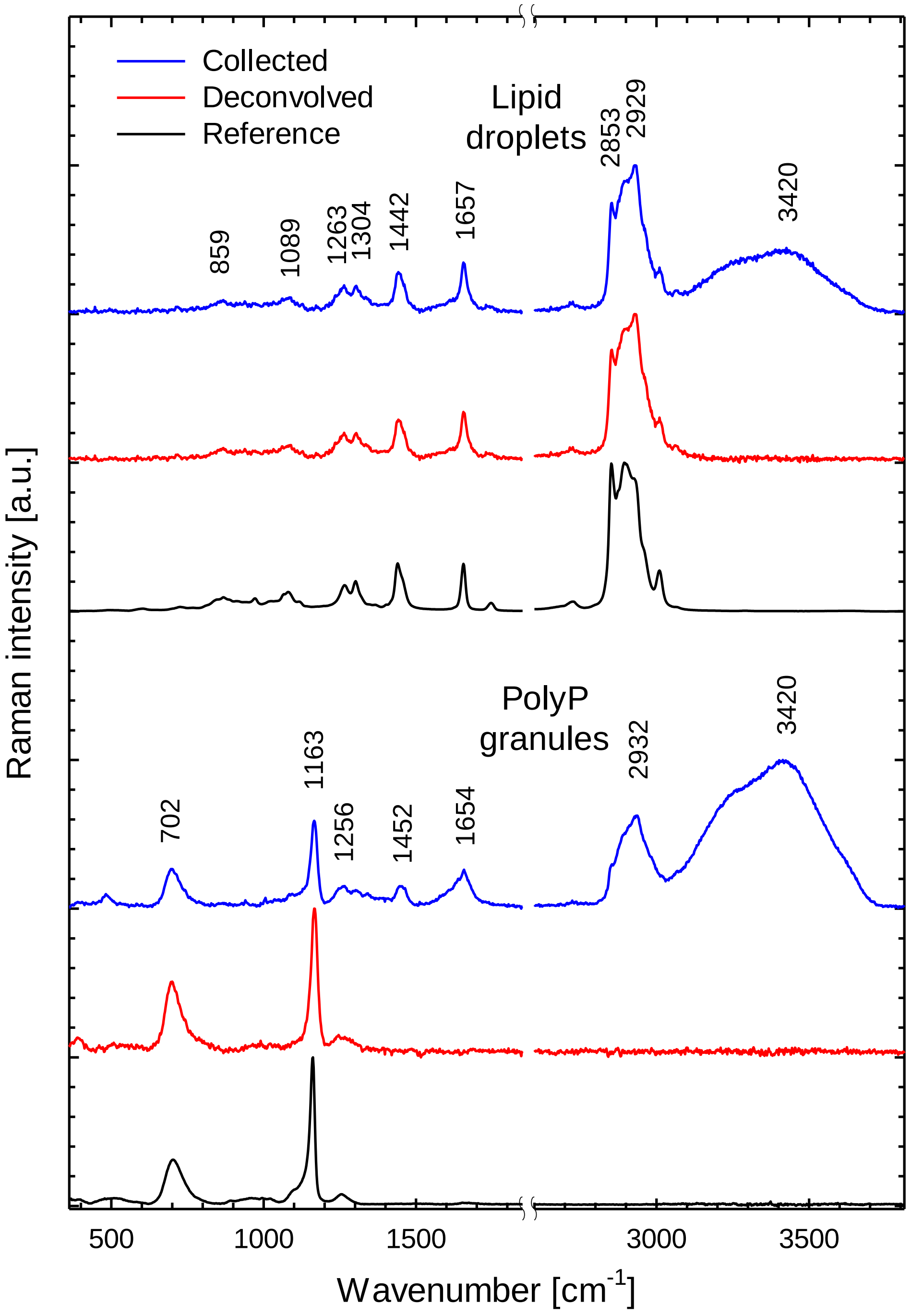

Figure S3: Typical Raman spectra of lipid droplets and polyphosphate granules acquired directly from the cellular structures consisting mainly of the respective chemical compound (Collected, blue lines) compared with the Raman spectra after multivariate deconvolution (Deconvolved, red lines) and the Raman spectra of the corresponding chemically-pure references (Reference, black lines). In the case of very small lipid droplets, the contribution of water from the surrounding cytoplasm is often visible (band at 3420 cm^{-1}). In the case of polyP, surrounding water, as well as lipids from the membrane envelope, contribute to the collected spectra (bands at 1452, 1654 and 2932 cm^{-1}). If polyP granules are tightly co-localized with guanine crystals, the collected Raman spectrum contains also guanine features.

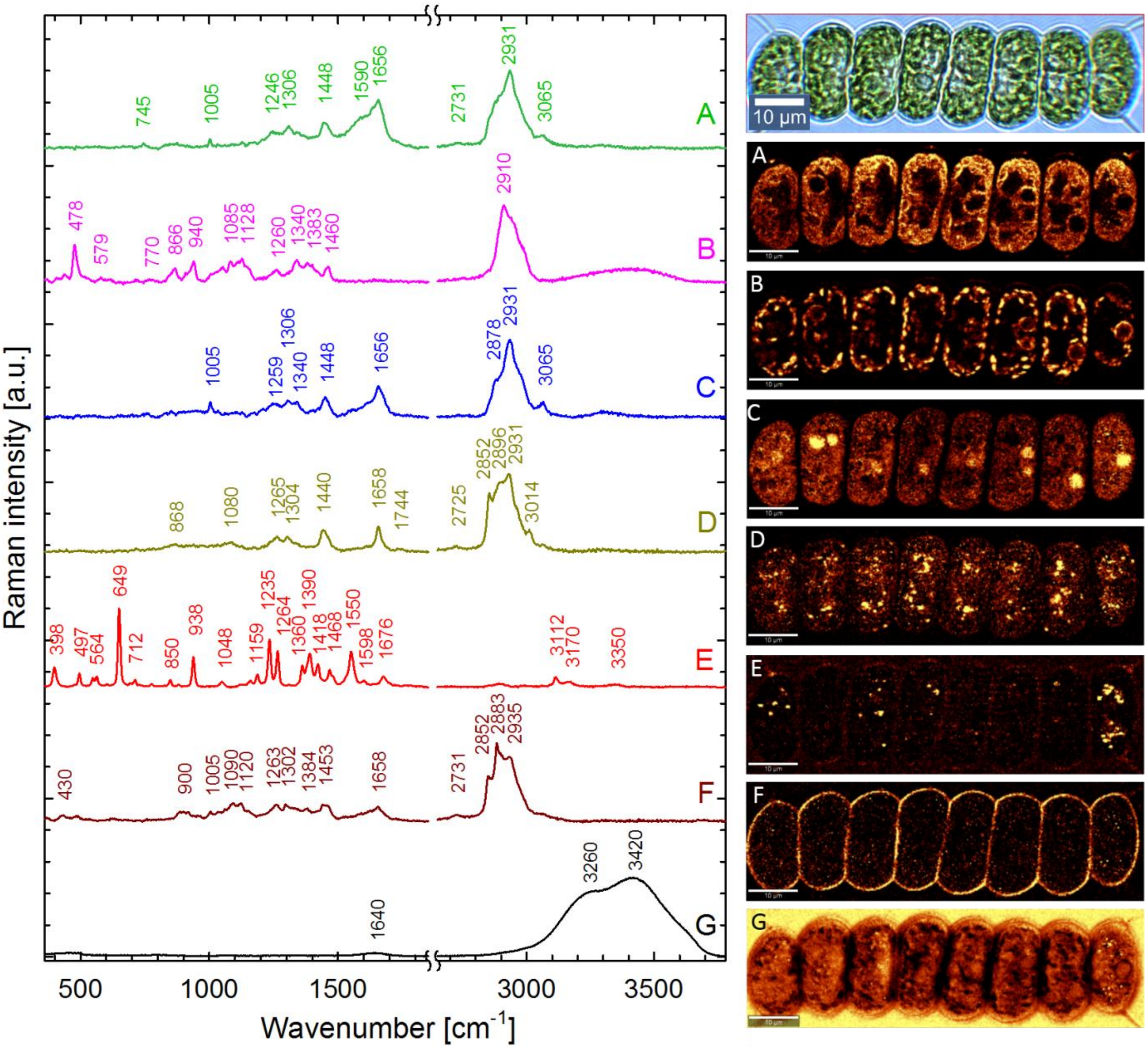

Figure S4: Bright-field image (top right) and Raman chemical maps of eight-cell coenobium of *D. quadricauda*. The hyperspectral dataset was decomposed into seven linearly independent spectral components assigned to plastids (A), starch (B), protein component coinciding with pyrenoids (C), neutral lipids (D), crystalline guanine (E), cell wall containing cellulose and membrane lipids (F) and water (G). The labeling of Raman spectra (A - H) on the left panel is the same as the labeling of the corresponding Raman chemical maps shown on the right panels. In this coenobium, the polyP was not found. For deconvolution, a multivariate analysis of the entire spectra was used. The left panel presents the resulting spectral components resulting directly from the multivariate analysis. To obtain a satisfactory residual error, all seven components were necessary.

Figure S5 (next four pages): Raman maps showing the distribution of carbon-hydrogen (C-H) groups, starch, lipids, polyphosphate and guanine in two innermost cells of eight-celled coenobia during the cell cycle. The time counted from the start of the light period (T = 00:00 h) that was understand as the beginning of the cell cycle, is indicated in the top row. For a given biomolecule, the color scale is the same for all the maps. The figure is the same as Fig. 1C, except for all the cells that were measured are shown. Due to a mechanical error of the microscope stage, only a part of the cells at T = 14:00 h was measured. At T = 17:45 h and T = 21:15 h, two coenobia of the same sample were used for measurement. The white bars correspond to 2 μm.

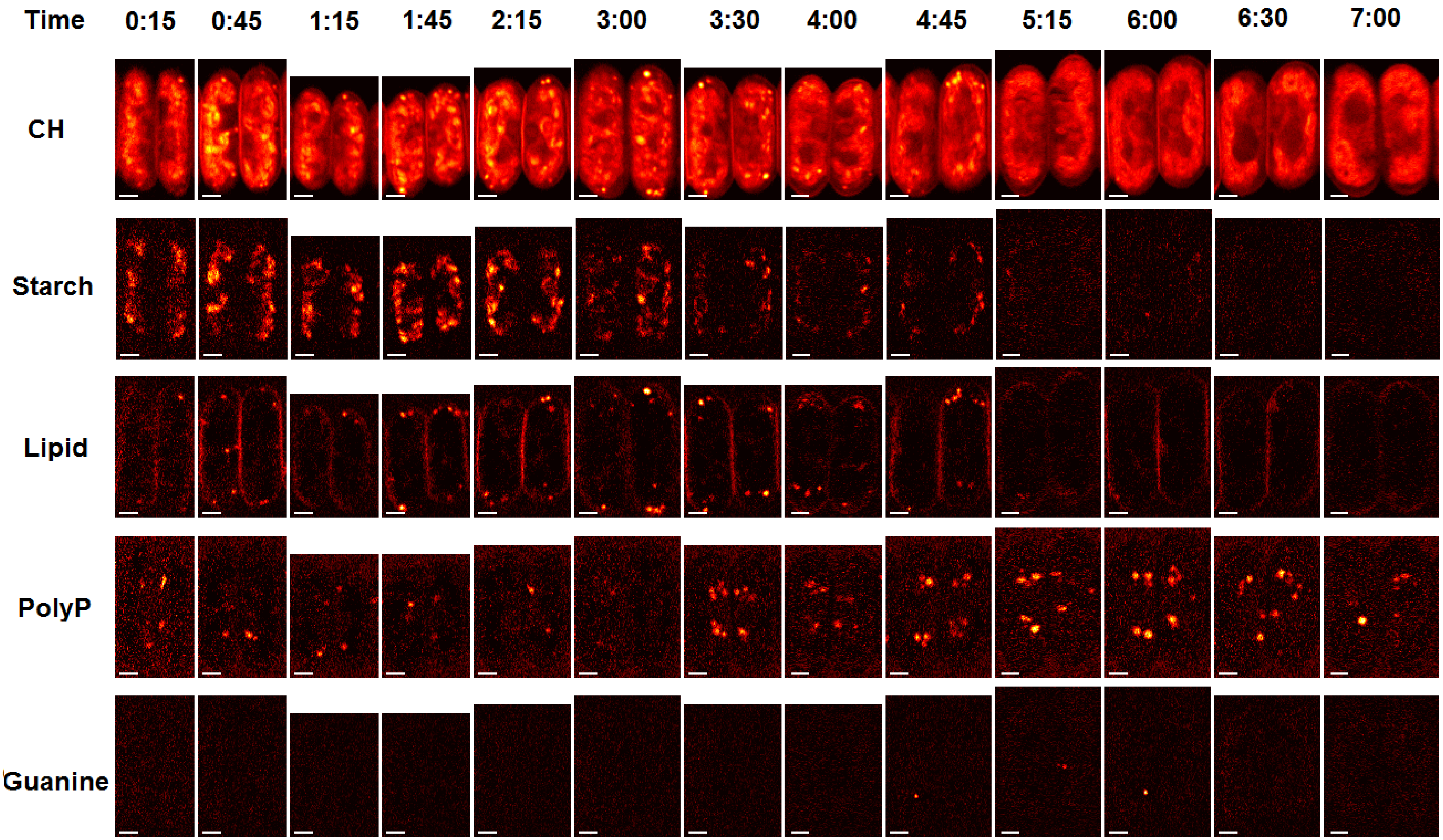
Time
0:15
0:45
1:15
1:45
2:15
3:00
3:30
4:00
4:45
5:15
6:00
6:30
7:00
CH
Starch
Lipid
PolyP
Guanine

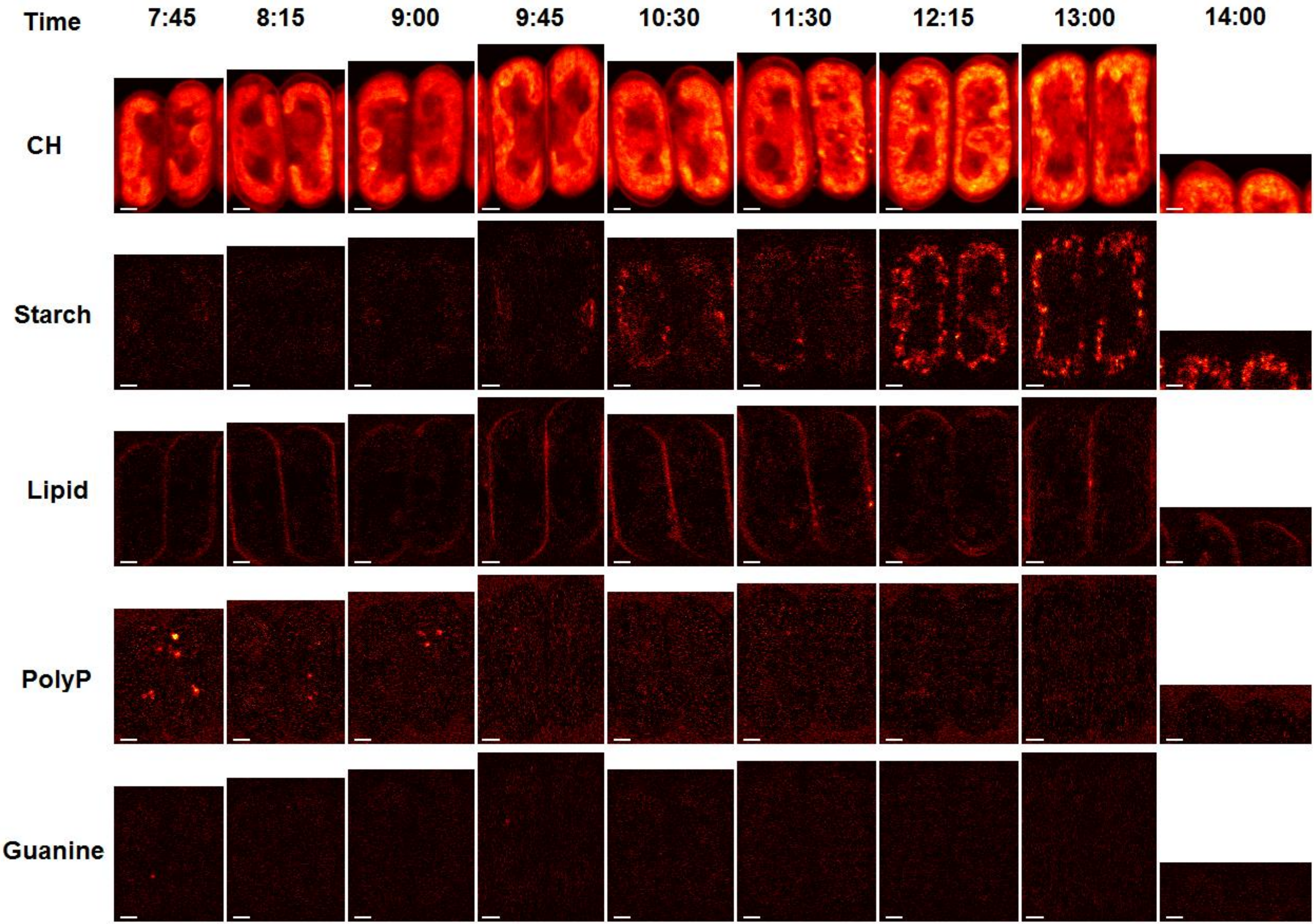

Time
7:45
8:15
9:00
9:45
10:30
11:30
12:15
13:00
14:00
CH
Starch
Lipid
PolyP
Guanine

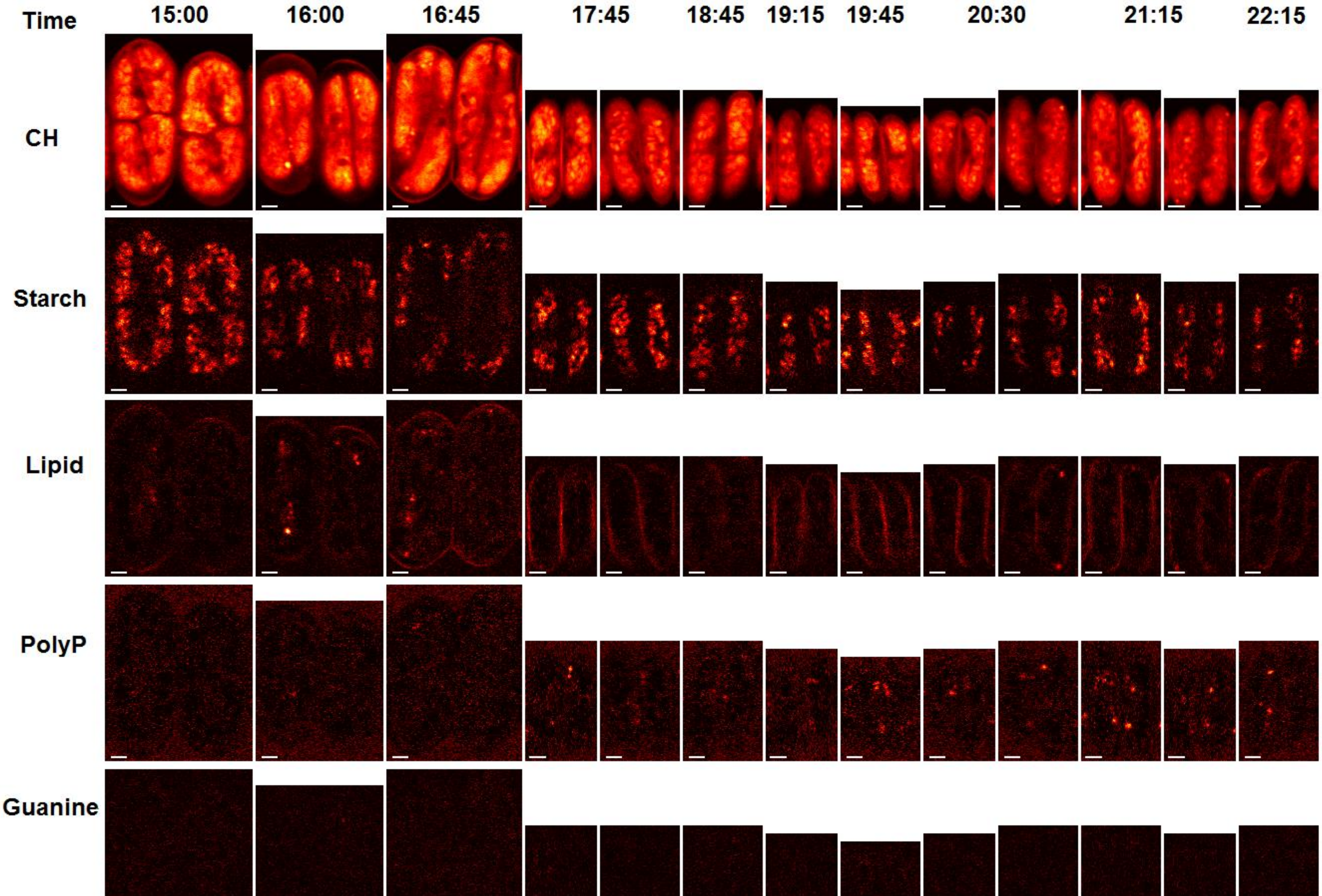
Time
15:00
16:00
16:45
17:45
18:45
19:15
19:45
20:30
21:15
22:15
CH
Starch
Lipid
PolyP
Guanine

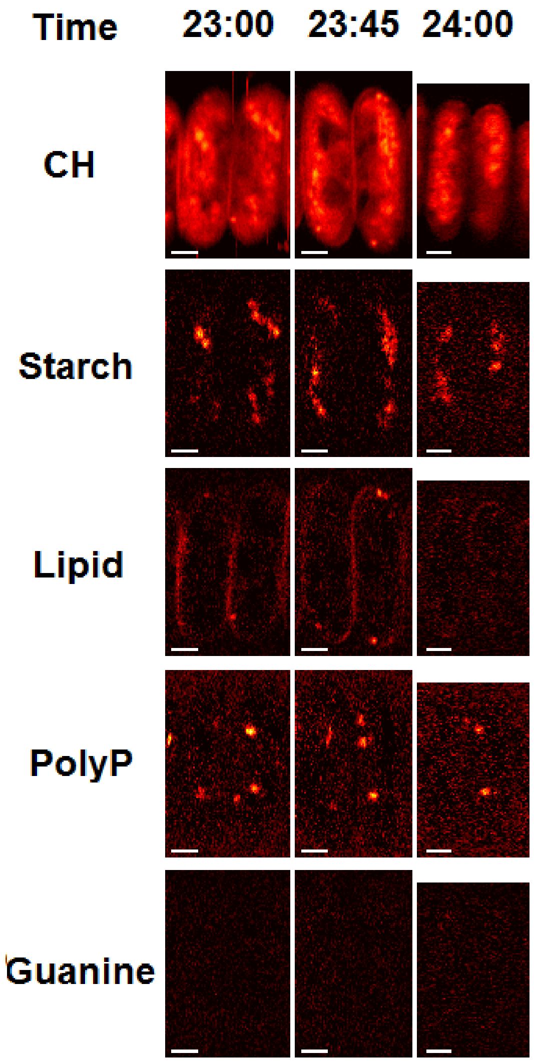
Time
23:00
23:45
24:00
CH
Starch
Lipid
PolyP
Guanine
